# Insulin resistance and body mass index are associated with TSPO PET in cognitively unimpaired elderly

**DOI:** 10.1177/0271678X231172519

**Published:** 2023-04-27

**Authors:** Laura L Ekblad, Jouni Tuisku, Mikko Koivumäki, Semi Helin, Juha O Rinne, Anniina Snellman

**Affiliations:** 1 Turku PET Centre, University of Turku and Turku University Hospital, Turku, Finland; 2 InFLAMES Reseach Flagship Center, University of Turku, Turku, Finland

**Keywords:** *APOE* genotype, PK11195, Alzheimer, TSPO, metabolic risk factor

## Abstract

Metabolic risk factors are associated with peripheral low-grade inflammation and an increased risk for dementia. We evaluated if metabolic risk factors i.e. insulin resistance, body mass index (BMI), serum cholesterol values, or high sensitivity C-reactive protein associate with central inflammation or beta-amyloid (Aβ) accumulation in the brain, and if these associations are modulated by *APOE4* gene dose. Altogether 60 cognitively unimpaired individuals (mean age 67.7 years (SD 4.7); 63% women; 21 *APOE3/3*, 20 *APOE3/4* and 19 *APOE4*/*4*) underwent positron emission tomography with [^11^C]PK11195 targeting TSPO (18 kDa translocator protein) and [^11^C]PIB targeting fibrillar Aβ. [^11^C]PK11195 distribution value ratios and [^11^C]PIB standardized uptake values were calculated in a cortical composite region of interest typical for Aβ accumulation in Alzheimer’s disease. Associations between metabolic risk factors, [^11^C]PK11195, and [^11^C]PIB uptake were evaluated with linear models adjusted for age and sex. Higher logarithmic HOMA-IR (standardized beta 0.40, p = 0.002) and BMI (standardized beta 0.27, p = 0.048) were associated with higher TSPO availability. Voxel-wise analyses indicated that this association was mainly seen in the parietal cortex. Higher logarithmic HOMA-IR was associated with higher [^11^C]PIB (standardized beta 0.44, p = 0.02), but only in *APOE4/4* homozygotes. BMI and HOMA-IR seem to influence TSPO availability in the brain.

## Introduction

Alzheimer's disease (AD) is the most prevalent form of dementia, and *APOE* ε4 (*APOE4*) genotype is the most important genetic risk factor for sporadic AD.^1,2^ In addition to the well-established association between *APOE4* and AD, the *APOE* gene also plays a role in vascular diseases and metabolic risk factors and, in turn, metabolic risk factors are acknowledged risk factors for AD.^3,4^ Carrying at least one *APOE4* allele increases the risk for coronary artery disease^5–7^ and is associated with elevated circulating low-density lipoprotein cholesterol (LDL-C) and total cholesterol levels,^6^ when compared to *APOE* ε3/ε3 (*APOE3/3*) genotype. In contrast, carrying the *APOE4* allele may be associated with a *decreased* risk for type 2 diabetes and obesity.^7,8^ Potentially modifiable metabolic risk factors measured in midlife such as hypertension,^9^ hypercholesterolemia,^10^ obesity,^11^ type 2 diabetes,^12,13^ and insulin resistance^14^ increase the risk for late-life dementia.^3^ However, the interplay between these risk factors, *APOE* genotype and cerebral changes related to AD is still unclear.

*APOE4* increases the risk for beta-amyloid (Aβ) accumulation, the first neuropathologic hallmark of AD, across the AD spectrum.^15^ We have previously shown that midlife insulin resistance was associated with higher Aβ-accumulation in late-life, and that this association is stronger in *APOE4* carriers than non-carriers.^16^ Neuropathological studies have demonstrated that the accumulation of Aβ-plaques in the cerebral cortex is accompanied with an inflammatory response, that is, microglia that are found in the different forms of Aβ-plaques (i.e. diffuse non-neuritic/diffuse neuritic/dense-core neuritic),^17^ and astrocytes that are found in the immediate vicinity of Aβ-plaques.^18^ Astrocytes are the main site of production of ApoE, the protein coded by *APOE*, in the brain.^19^ This protein is produced in three different isoforms: ApoE2, ApoE3 and ApoE4, depending on which of the *APOE* allelles are present, and the different ApoE isoforms likely contribute to the different risk profiles for AD and other diseases associated with the *APOE* polymorphism.^6,20^ Moreover, *APOE4* gene dose (the number of ε4 alleles) has been shown to correlate with the amount of activated microglia in AD brains, indicating that *APOE* genotype might also play a direct role in microglial activation.^21^ However, very little is known about the association between metabolic risk factors and the reaction of microglia and astrocytes to underlying pathological processes. Thus, it is important to study the interplay among metabolic risk factors, *APOE* genotype, Aβ-accumulation, and central inflammation to better understand the complex role of metabolic risk factors and *APOE* in AD.

PET targeted at detecting neuroinflammation *in vivo* is mostly based on radiotracers that bind to 18-kDa translocator protein (TSPO) that is expressed on the outer mitochondrial membrane of activated microglia.^22^ These tracers also bind to other sites than microglia, such as the endothelium and astrocytes.^23^ Nevertheless, some previous studies indicate that TSPO-binding is greater in AD patients in certain brain regions when compared to cognitively normal controls and mild cognitive impairment (MCI) patients.^24–26^ However, our previous work suggests that TSPO-binding, measured with a second-generation tracer [^11^C]PBR28, may be associated with metabolic risk factors such as body mass index (BMI)^27,28^ and homeostasis model of insulin resistance (HOMA-IR).^27^ We have recently demonstrated that although there was a clear difference in Aβ-accumulation, measured with [^11^C]PIB-PET, no difference in TSPO availability, measured with the most widely used TSPO-radiotracer [^11^C]PK11195, was found among cognitively unimpaired *APOE3/3*, *APOE3/4*, and *APOE4/4* carriers.^29^

Based on the previous literature, we hypothesized that metabolic risk factors (insulin resistance, BMI, serum cholesterol values or high sensitivity C-reactive protein) may be associated with TSPO availability and Aβ-accumulation already in cognitively unimpaired individuals. To test this hypothesis, we evaluated the associations between metabolic risk factors and [^11^C]PK11195 and [^11^C]PIB binding in brain regions typically affected by early Aβ-accumulation in a cohort of cognitively unimpaired elderly (n = 60). In addition, we had a secondary hypothesis, that *APOE* genotype might modulate these associations. For this more exploratory hypothesis, we performed additional analyses separately in *APOE3/3* (n = 21), *APOE3/4* (n = 20) and *APOE4/4* (n = 19) carriers.

## Methods

### Study population and recruitment

ASIC-E4 is a cross-sectional, multimodal neuroimaging study that was set out to explore gene dose effects of *APOE4* on AD-related cerebral changes by recruiting individuals who were non-carriers (*APOE3/3*, n = 20), heterozygotes (*APOE3/4*, n = 21) or homozygotes (*APOE4/4*, n = 19) for the ε4 allele. The details of the study protocol and recruitment have been previously published.^30^ The participants were recruited via the local AURIA biobank according to age (60 to 75 years) and *APOE* genotype. All participants were community-dwelling and did not require any assistance with their activities of daily living. The Finnish version of the CERAD (Consortium to Establish a Registry for Alzheimer´s Disease) test battery was performed at the screening visit, and those with a CERAD total score below 2 standard deviations (SD) (<62) of the elderly Finnish population were excluded. Further exclusion criteria were any major neurological or psychiatric disease; diabetes; any chronic inflammatory condition such as rheumatoid arthritis; use or corticosteroids; and any contraindication for a PET or MRI scan, such as claustrophobia or a metal object in the body. Three participants did not complete [^11^C]PK11195 imaging and thus, 57 individuals were included in the [^11^C]PK11195 analyses of the present study. The main aim of the ASIC-E4 study is to investigate *APOE4* gene dose effects on AD-related cerebral changes and blood biomarkers.^30^ This study is a sub-study utilizing the data collected from this cohort, where associations between the obtained imaging and metabolic variables are evaluated primarily in the whole cognitively unimpaired study sample.

## Standard protocol approvals, registrations, and patient consents

The ASIC-E4 study was approved by the Ethics Committee of the Hospital District of Southwest Finland. The study was carried out according to the World Medical Association Declaration of Helsinki. All participants signed a written informed consent before being enrolled in the study.

## Brain MRI and PET imaging

Structural T1-weighted brain MRI scan was performed with either Philips Ingenuity 3.0 T TF PET-MR (Philips Healthcare, Amsterdam, the Netherlands), or Philips Ingenia 3.0 T (Philips Healthcare, Amsterdam, the Netherlands). The [^11^C]PIB- and [^11^C]PK11195-PET scans were acquired on an ECAT high-resolution research tomograph (HRRT, Siemens Medical Solutions, Knoxville, TN). The [^11^C]PIB scans were acquired 40 to 90 minutes post injection. The [^11^C]PK11195 scans were dynamic and were acquired 0 to 60 minutes post injection. All images were reconstructed with 3D ordinary Poisson ordered subset expectation maximization algorithm (OP-OSEM3D), and list mode data was histogrammed into 8 (6 × 5 + 2 × 10 min) and 17 (2 × 15; 3 × 30; 3 × 60; 7 × 300; 2 × 600 s) timeframes, respectively.

## Brain imaging analysis

An automated neuroimage analysis pipeline at Turku PET Centre^31^ was used for preprocessing of imaging data. [^11^C]PIB binding was quantified as standardized uptake value ratios (SUVR) calculated for 60 to 90 minutes post injection, using the cerebellar cortex as a reference region, and [^11^C]PK11195 binding as distribution volume ratios (DVR) using a reference tissue input Logan’s method within 20–60 min, where supervised clustering algorithm utilized to calculate the pseudo-reference region.^32,33^ The kinetic classes for supervised clustering were defined from a separate HRRT dataset from our center.^34^ Kinetic classes describing binding in normal GM, normal WM, and vasculature were defined using data from control subjects (n = 8, mean age 49.7, (SD 10.5) years; age range, 39–66 years; 6 women and 2 men). In the present study, no differences in the reference region SUVs among the three *APOE* genotypes were observed (ANOVA for *APOE* genotype group differences p = 0.68). To minimize the effect of TSPO binding to cerebral sinuses, both regional and voxel level [^11^C]PK11195 data was corrected for partial volume effects with PETPVE12 toolbox in SPM12, where geometric transfer matrix method was applied for regional data and Müller-Gärtner method for voxel level data as described previously.^30^ A cortical composite region-of-interest (ROI) was formed from prefrontal cortex, parietal cortex, anterior cingulum, posterior cingulum, precuneus and lateral temporal cortex (based on regions of early amyloid accumulation in AD),^35,36^ and a volume weighted composite of these ROIs was calculated. Normalized parametric [^11^C]PIB SUVR and [^11^C]PK11195 binding potential (BP_ND_) images smoothed using Gaussian 8 mm FWHM filter were used for all voxel-wise statistical analysis. Aβ positivity was defined as cortical composite [^11^C]PIB SUVR > 1.5, based on previous studies on healthy elderly populations.^37,38^

## Study examinations and laboratory measurements

Fasting venous blood samples were drawn from all participants after an overnight fast (10-12 h). Serum high-sensitivity C-reactive protein (hs-CRP), plasma total cholesterol, LDL-C, high density lipoprotein cholesterol (HDL-C), triglycerides, plasma insulin, and plasma glucose were measured as reported previously.^30^ Homeostatic model assessment of insulin resistance (HOMA-IR) was calculated as: (fasting insulin (μU/mL) x fasting glucose (mmol/L))/22.5.^39^ Weight and height were measured at the screening visit and BMI was calculated as weight in kilograms divided by (height (in meters)).^2^

## Statistical analysis

Normality of the distribution of all variables was inspected from the histograms and evaluated with Shapiro-Wilk’s test. A logarithmic (log_e_) transformation was performed for the variables that were skewed to achieve a normal distribution (HOMA-IR, hs-CRP, HDL-C). Differences in group demographics between the three gene doses were tested using one-way ANOVA or Kruskall-Wallis test with Tukey's honest significance test/the Steel-Dwass method for multiple comparisons for continuous variables, and χ^2^ test for categorical variables.

To test the primary hypothesis, correlations between the metabolic risk factors (logarithmic HOMA-IR, BMI, logarithmic hs-CRP, total cholesterol, LDL-C and logarithmic HDL-C) and [^11^C]PK11195 DVRs in the cortical composite ROI in the total study population were first analyzed with Pearson’s correlation. [^11^C]PIB SUVRs in the total study population did not follow a normal distribution, and therefore correlations between metabolic risk factors and [^11^C]PIB SUVR cortical composite score were analyzed with Spearman’s correlation. Subsequently, associations between the variables that showed a significant correlation were further evaluated with linear regression models adjusted for age and sex. Additionally, all analyses were further adjusted for education.

To test the secondary hypothesis (possible modulating effect of *APOE* genotype), the interaction of ‘*APOE* genotype × logarithmic HOMA-IR’ and ‘*APOE* genotype × BMI’ were analyzed for the associations with [^11^C]PK11195 DVR and [^11^C]PIB SUVR in unadjusted models. Exploratory analyses were also performed separately for each *APOE* genotype group according to visual inspection of the regression plots (Figure 2). [^11^C]PIB SUVR in each of the *APOE* genotype group did not follow a normal distribution, and therefore all correlations were assessed with Spearman’s correlation. Stratified linear regression models adjusted for age and sex were performed for the variables showing significant correlations.

To explore possible interactions for sex on the associations between metabolic risk factors and TSPO availability and Aβ accumulation, the interaction of ‘sex × logarithmic HOMA-IR’ and ‘sex × BMI’ were analyzed for the associations with [^11^C]PK11195 DVRs and [^11^C]PIB SUVRs in unadjusted models. Analyses stratified for sex were performed if the interaction effect was significant.

Normality assumption of the of linear models were inspected from the residuals for all models. The analyses were performed with SAS JMP 16.0 (Cary, North Carolina, U.S.A.). P-value <0.05 (two-tailed) was considered statistically significant for all analyses.

Voxel-based associations between [^11^C]PK11195 binding and BMI, and [^11^C]PK11195 binding and logarithmic HOMA-IR were tested with linear regression with statistical parametric mapping (SPM), to evaluate if these associations were present also outside the *a priori* chosen ROIs. P < 0.001 was considered statistically significant in the voxel-based analyses, and false discovery rate (FDR) correction for cluster threshold was used to correct for multiple comparisons.

## Data availability

Anonymized data can be shared within reasonable request for a study plan that has been approved by a local ethics committee.

## Results

### Population characteristics

The mean age of the total study population was 67.6 (SD 4.7) years and 63.3% (n = 38) were women. The characteristics in the total population are shown in Table 1. Altogether 34 individuals (57%) were considered PIB positive according to the cut-off >1.5 [^11^C]PIB SUVR in the cortical composite ROI. Altogether 22 (36.7%) had medication for hypercholesterolemia. No significant differences between the *APOE* groups were detected in BMI (p = 0.86) or HOMA-IR (p = 0.44). *APOE4* homo- and heterozygotes had numerically higher mean total cholesterol (5.2 and 5.3 mmol/L vs 4.8 mmol/L) and LDL-C levels (3.3 and 3.3 mmol/L vs 3.0 mmol/L) when compared to *APOE3/3* homozygotes, but the differences were not statistically significant (p > 0.21 for both). Significantly lower median hs-CRP levels were present in *APOE4/4* homozygotes in comparison to non-carriers (p = 0.044), although all hs-CRP measurements were within the given reference values (upper limit 2.5 for women and 3.5 for men). (Table 2)

**Table 1.** Characteristics of the total study population.

	Mean/median	SD/interquartile range
n	60	
n for PK11195	57	
Age (y)	67.6	4.7
Sex (M/F), n (%)	22/38 (37/63)	
Education, n (%)		
Primary school	18 (30)	
Middle or comprehensive school	11 (18)	
High school	20 (33)	
College or university	11 (18)	
CERAD total score	85.4	8.2
BMI (kg/m^2^)	26.9	4.3
Total cholesterol (mmol/L)	5.06	0.97
LDL-C (mmol/L)	3.21	0.78
HDL-C (mmol/L)	1.63	1.38–1.85
Medication for hypercholesterolemia, n (%)	22 (37)	
Medication for hypertension, n (%)	26 (43)	
hs-CRP (mg/L)	0.90	0.50–1.75
HOMA-IR	2.01	1.21–3.45
PIB positivity, n (%)	34 (57)	
[^11^C]PK11195 composite DVR, n = 57	1.33	0.06
[^11^C]PIB composite SUVR	1.61	1.43–2.14

The data are presented as mean and standard deviation or median and interquartile range, unless stated otherwise. BMI: body mass index; CERAD; Consortium to establish a Registry for Alzheimer’s disease; DVR: distribution volume ratio; LDL-C: low-density lipoprotein cholesterol; HDL-C: high density lipoprotein cholesterol; hs-crp: high-sensitivity C-reactive protein; SUVR: standardized uptake value ratio. Three individuals did not complete [^11^C]PK11195 imaging.

**Table 2.** Metabolic risk factors according to *APOE* genotype.

	APOE3/3	APOE3/4	APOE4/4	p-value
n	20	21	19	
n for PK11195	17	21	19	
BMI (kg/m^2^)	27.3 (5.0)	26.7 (3.5)	26.6 (4.5)	0.86
Total cholesterol (mmol/L)	4.75 (0.98)	5.27 (0.79)	5.16 (1.11)	0.21
LDL-C (mmol/L)	3.01 (0.89)	3.32 (0.63)	3.31 (0.81)	0.36
HDL-C (mmol/L)	1.63 (1.39–1.77)	1.62 (1.47–1.91)	1.63 (1.18-1.91)	0.80
Medication for hypercholesterolemia, n (%)	6 (30)	7 (33)	9 (47)	0.50
Medication for hypertension, n (%)	9 (45)	8 (38)	9 (47)	0.82
hs-CRP (mg/L)	1.40 (0.73–2.08)	0.90 (0.50–1.95)	0.70 (0.20–1.10)*	0.044
HOMA-IR	2.14 (1.23–4.40)	1.90 (1.19–2.56)	2.32 (1.24–3.53)	0.44

The data are presented as mean (standard deviation) or median (interquartile range), unless stated otherwise. P-values for overall differences between *APOE* groups, assessed with ANOVA (normally distributed variables)/Kruskall-Wallis test (skewed variables)/χ^2^ -test (categorical variables). Pairwise comparisons were assessed with Tukey’s honest significance test for normally distributed variables and the Steel-Dwass method for skewed variables.

* p < 0.05 in pairwise comparisons (Tukey HSD) compared with to *APOE3/3*. Three individuals from the *APOE3/3* group did not complete [^11^C]PK11195 imaging. BMI: body mass index; LDL-C: low-density lipoprotein cholesterol; HDL-C: high density lipoprotein cholesterol; hs-crp: high-sensitivity C-reactive protein.

## Correlations between metabolic risk factors and [^11^C]PK11195 in the total study population

**Figure 1.**
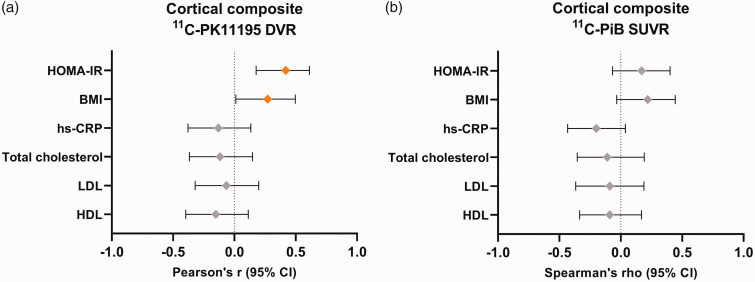
Correlation coefficients and 95% confidence intervals for the associations between metabolic risk factors and (a) cortical composite [^11^C]PK11195 distribution volume ratio (DVR) and (b) cortical composite [^11^C]PIB standardized uptake value ratio (SUVR) in the total study population. The analyses for [^11^C]PK11195 were performed with Pearson’s correlation and for [^11^C]PIB SUVR with Spearman’s correlation due to the skewed distribution of [^11^C]PIB SUVR. A logarithmic transformation was used for HOMA-IR, hs-CRP and HDL-C. LDL-C: low-density lipoprotein cholesterol; HDL-C: high density lipoprotein cholesterol; HOMA-IR: homeostasis model of insulin resistance; hs-crp: high-sensitivity C-reactive protein. n = 57 for [^11^C]PK11195 and n = 60 for [^11^C]PIB.

Both higher logarithmic HOMA-IR (r = 0.42, p = 0.001) and higher BMI (r = 0.27, p = 0.04) correlated with a higher cortical composite [^11^C]PK11195 DVR in the total study population, whereas no associations between logarithmic hs-CRP, total cholesterol, LDL-C or logarithmic HDL-C and [^11^C]PK11195 were found (r between −0.15 and −0.06, p > 0.26 for all, Figure 1(a)). The correlation between logarithmic HOMA-IR and [^11^C]PK11195 was significant even after Bonferroni correction (Bonferroni corrected p-value for statistical significance: p = 0.05/6 = 0.008), whereas the associations between BMI and [^11^C]PK11195 did not survive correction for multiple comparisons.

## Correlations between metabolic risk factors and [^11^C]PIB in the total study population

Metabolic variables did not show significant correlations with cortical composite [^11^C]PIB SUVR in the total study population (rho between −0.20 and 0.17, p > 0.13 for all, Figure 1(b)).

## Linear models adjusted for age and sex in the total study population

Based on the correlation analyses presented above, the association between metabolic risk factors and [^11^C]PK11195 DVR were analyzed also with linear regression models adjusted for age and sex for the variables with significant correlations. Higher logarithmic HOMA-IR (standardized β = 0.40, p = 0.002) and BMI (standardized β = 0.27, p = 0.049) were associated with higher [^11^C]PK11195 DVR in the cortical composite ROI. Age and sex were not associated with [^11^C]PK11195 DVR in our cohort (Table 3). Additional analyses that were further adjusted for education were also performed. Education was not associated with [^11^C]PK11195 DVR (p = 0.87).

**Table 3.** Associations between logarithmic HOMA-IR and BMI and cortical composite [^11^C]PK11195 binding in the total study population.

	Cortical composite [^11^C]PK11195 DVR in the total population (n = 57)			
	β	Confidence interval	Std β	Adjusted R^2^ (%)
Whole model				14.4
log_HOMA-IR	0.04	0.02 to 0.07**	0.40	16.0
Age	0.001	−0.003 to 0.004	0.05	−1.6
Sex	0.006	−0.01 to 0.02	0.10	1.7
Whole model				5.3
BMI	0.004	0.00002 to 0.008*	0.27	5.7
Age	0.0007	−0.003 to 0.004	0.05	−1.6
Sex	0.010	−0.008 to 0.028	0.15	1.7

The data are presented as estimates (β), confidence intervals and standardized estimates (std β) for each variable in the linear regression model. Logarithmic HOMA-IR and BMI were analyzed in separate models, adjusted for age and sex. Adjusted R^2^ indicates the explanatory value of the whole model, and of each variable when entered into the model alone, without the covariates. *p < 0.05, **p < 0.01.

## Voxel-wise regression analyses

**Figure 2.**
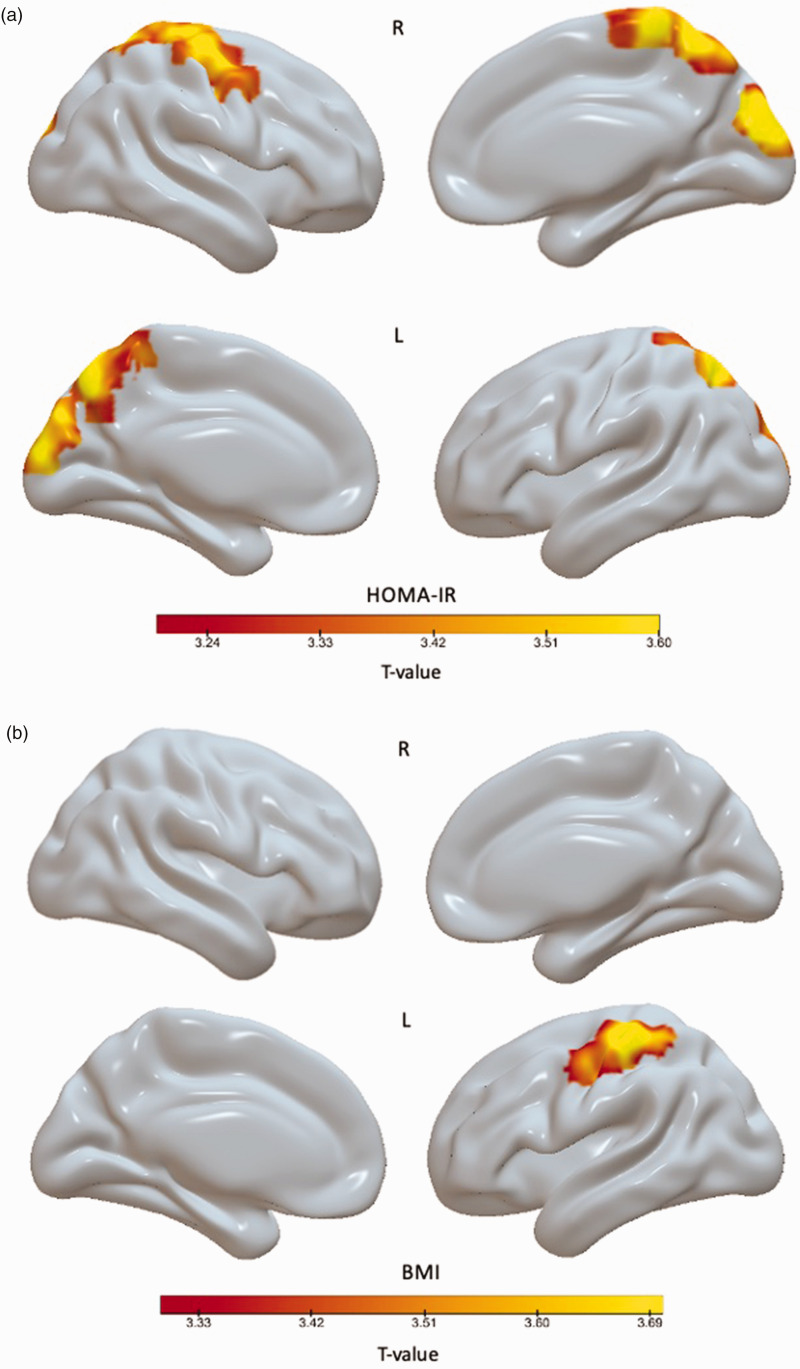
Voxel-wise regression analysis of the association between [^11^C]PK11195 and BP_ND_ and (a) logarithmic HOMA-IR and (b) body mass index (BMI) in the total study population. Figure 2(a) indicates voxels where higher logarithmic HOMA-IR showed a significant association with higher [^11^C]PK11195 BP_ND_ on statistical parametric mapping analyses (thresholded combining p < 0.001 at voxel level and false discovery rate (FDR) corrected p < 0.05 at cluster level). Figure (b) indicates voxels where higher BMI showed a significant association with higher [^11^C]PK11195 BP_ND_ (thresholded combining p < 0.001 at voxel level and FDR corrected p < 0.05 at cluster level). Brighter colors indicate a stronger correlation.

Voxel-wise analyses in the total cognitively unimpaired study population showed that the association between logarithmic HOMA-IR and [^11^C]PK11195 was mostly found in the parietal cortex/the precuneus and the occipital cortex (Figure 2(a)). The association between BMI and [^11^C]PK11195 was restricted to the left parietal cortex (Figure 2(b)).

## Exploratory results: analyses stratified according to *APOE* genotype

**Figure 3.**
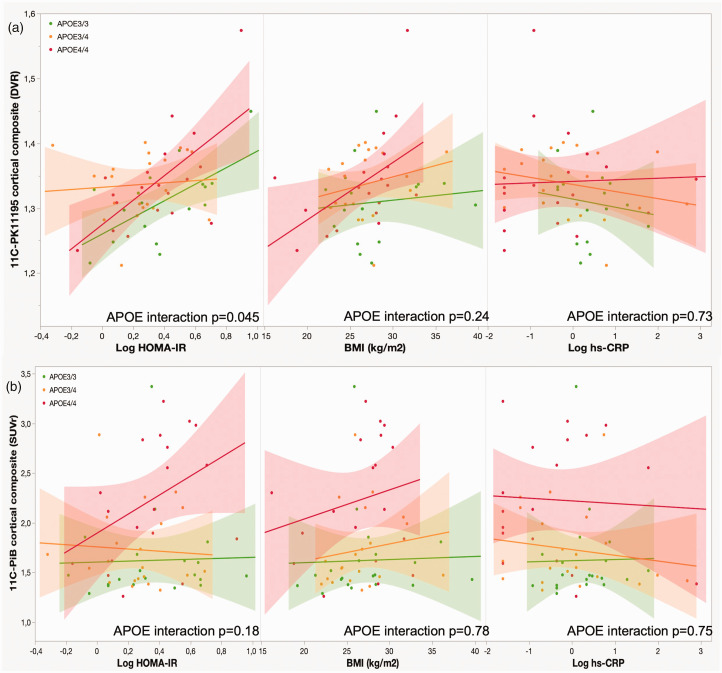
Associations between metabolic risk factors and (a) cortical composite [^11^C]PK11195 distribution volume ratio (DVR) and (b) cortical composite [^11^C]PIB standardized uptake value ratio (SUVR). The figure represents scatter plots with regression lines and 95% confidence intervals according to *APOE* genotype for the associations between metabolic risk factors and (a) cortical composite [^11^C]PK11195 distribution volume ratio (DVR) and (b) cortical composite [^11^C]PIB standardized uptake value ratio (SUVR). P-values for the interaction for APOE genotype and each metabolic variable [^11^C]PK11195 DVR and [^11^C]PIB SUVR are derived from unadjusted linear models.

A significant interaction for ‘*APOE* genotype × logarithmic HOMA-IR’ (p = 0.045) was found on the association with cortical composite [^11^C]PK11195 DVR (Figure 3). When correlations were then explored within each *APOE* genotype, a positive correlation was found in *APOE4/4* (r = 0.65, p = 0.003) and *APOE3/3* (r = 0.63, p = 0.007) homozygotes, whereas no correlation was present in *APOE3/4* carriers (r = 0.04, p = 0.86). There was no significant interaction for *APOE* genotype with BMI on the association with cortical composite [^11^C]PK11195 DVR (p = 0.24).

There was no significant “*APOE* genotype × logarithmic HOMA-IR” (p = 0.18) or “*APOE* × BMI” (p = 0.78) interaction on the association with the composite cortical [^11^C]PIB SUVR (Figure 3). However, visual inspection of the regression plots suggested that the correlation between logarithmic HOMA-IR and cortical [^11^C]PIB SUVR would be strongest in *APOE4/4* homozygotes. An additional exploratory analysis showed a borderline significant correlation between higher logarithmic HOMA-IR and a higher [^11^C]PIB SUVR composite score in the *APOE4*/*4* group (rho = 0.44, p = 0.06), whereas there was no association in *APOE3/3* (rho = 0.16, p = 0.50) or *APOE3/4* (rho = −0.052, p = 0.82) carriers.

In line with the correlation analyses, the linear models adjusted for age and sex indicated that logarithmic HOMA-IR was associated with cortical composite [^11^C]PK11195 DVR in *APOE3/3* (standardized β = 0.56 p = 0.02) and *APOE4/4* (standardized β = 0.60, p = 0.009) homozygotes, but not in *APOE3/4* (standardized β = 0.04, p = 0.87) carriers. Logarithmic HOMA-IR was associated with the cortical composite [^11^C]PIB SUVR in *APOE4/4* homozygotes (standardized β = 0.44, p = 0.02) in the model adjusted for age and sex. (Table 4) The association between logHOMA-IR and [^11^C]PIB SUVR remained significant even after adjustments for education (standardized β = 0.36, p = 0.03).

**Table 4.** Associations between logarithmic HOMA-IR and (A) cortical composite [^11^C]PK11195 DVR and (B) cortical composite [^11^C]PIB SUVR stratified by *APOE* genotype.

A	Composite cortical [^11^C]PK11195 DVR					
	*APOE3/3, n = 17*		*APOE3/4, n = 21*		*APOE4/4, n = 19*	
	β (CI)	std β	β (CI)	std β	β (CI)	std β
logHOMA-IR	0.05 (0.01 to 0.09)*	0.56	0.004 (−0.05 to 0.05)	0.04	0.08 (0.02 to 0.13)**	0.60
Age	0.001 (−0.004 to 0.007)	0.10	0.001 (−0.01 to 0.01)	0.06	0.0002 (−0.01 to 0.01)	0.01
Sex	0.01 (−0.01 to 0.04)	0.26	−0.01 (−0.04 to 0.02)	−0.19	0.01 (−0.02 to 0.05)	0.17
B	Composite cortical [^11^C]PIB SUVR					
	*APOE3/3, n = 20*		*APOE3/4, n = 21*		*APOE4/4, n = 19*	
	β (CI)	std β	β (CI)	std β	β (CI)	std β
logHOMA-IR	0.05 (−0.31 to 0.40)	0.07	−0.01 (−0.37 to 0.36)	−0.01	0.45 (0.09 to 0.80)*	0.44
age	−0.02 (−0.07 to 0.04)	−0.18	0.01 (−0.04 to 0.05)	0.09	0.09 (0.05 to 0.13)***	0.69
sex	−0.06 (−0.32 to 0.20)	−0.14	0.15 (−0.06 to 0.36)	0.36	−0.13 (−0.35 to 0.13)	−0.20

The data are presented as estimates (β), confidence intervals (CI) and standardized estimates (std β) for each variable in the linear regression model. *p < 0.05, **p < 0.01, ***p < 0.001.

*APOE* genotype did not modulate the associations between hs-CRP and [^11^C]PK11195 DVR or [^11^C]PIB SUVR (p > 0.73 for all) (Figure 3).

## Exploratory results: Interactions for sex

The interaction for ‘sex × logHOMA-IR’ on the association with composite cortical [^11^C]PK11195 DVR was significant (p = 0.003), whereas there was no interaction for ‘sex × BMI’ (p = 0.24). Stratified analyses suggested that the association between logarithmic HOMA-IR and [^11^C]PK11195 DVR was present in men, but not in women (unadjusted, men: standardized β = 0.76, p < 0.0001; women: standardized β = 0.04, p = 0.78; adjusted for age, men: standardized β = 0.77, p < 0.0001; women: standardized β = 0.05, p = 0.76). There were no interactions for sex on the associations between logHOMA-IR (p = 0.42) or BMI (p = 0.81) and cortical composite [^11^C]PIB SUVR.

## Discussion

Here, we demonstrate that in a sample of cognitive unimpaired elderly, higher levels of insulin resistance and BMI are associated with higher [^11^C]PK11195 binding in the cerebral cortex in regions typical for Aβ accumulation in AD. For insulin resistance, these associations were present in *APOE3/3* and *APOE4/4* carriers, but not in *APOE3/4* heterozygotes. No associations were found between hs-CRP or serum cholesterol values and TSPO binding, or between insulin resistance, BMI and [^11^C]PIB in the total study population. Higher HOMA-IR, a marker of insulin resistance, was associated with [^11^C]PIB only in *APOE4/4* homozygotes. Since diabetes was an exclusion criterion in the present study the results indicate that insulin resistance already before the onset of type 2 diabetes could play a role in the early pathological process of AD. In addition, the associations between HOMA-IR and [^11^C]PK11195 were modulated by sex: higher HOMA-IR was associated with higher cortical composite [^11^C]PK11195 in men, but not in women.

We have previously demonstrated similar associations between insulin resistance and BMI and TSPO binding mainly in the parietal cortex utilizing [^11^C]PBR28, a second-generation TSPO ligand, in a sample of elderly (mean age 70.1 years) individuals without dementia.^27^ The present study was performed on a different and slightly younger study population (mean age 67.6 years), that was enriched for *APOE4* homo- and heterozygotes, and the study protocol included screening for cognitive decline. Altogether 34 (56.7%) of the participants in the present study had an amyloid positive [^11^C]PIB-PET scan (cortical composite SUVR > 1.5), suggesting that these individuals had “Alzheimer´s pathologic change” or “preclinical AD”, and were in the Alzheimer´s continuum according to the A/T/N classification and the updated NIA-AA research criteria.^40,41^ In accordance with our previous study with [^11^C]PBR28, voxel-based analyses in the present study indicated that the associations between HOMA-IR and [^11^C]PK11195 were mostly restricted to the parietal cortex. The findings on association between HOMA-IR and Aβ accumulation in the brain in *APOE4/4* homozygotes are also in line with our previous findings which suggest that insulin resistance seems to be an additive risk factor for Aβ accumulation in *APOE4* carriers.^16^ In the present study population, a correlation between [^11^C]PK11195 and [^11^C]PIB cortical composites was previously detected in only in *APOE4/4* homozygotes.^29^ We did not find an association between hs-CRP or cholesterol values and TSPO binding or Aβ accumulation. These negative findings in this relatively small study population are not surprising, since hs-CRP levels can vary according to, for example, mild infections, and altogether 37% of the studied individuals were using lipid-lowering medication which influenced their total cholesterol, LDL-C and HDL-C values.

Although [^11^C]PK11195-PET imaging has been used in multiple studies focusing on patients with AD or mild cognitive impairment (MCI), these previous studies have not evaluated the possible associations between common metabolic risk factors and [^11^C]PK11195 binding. It appears that, similar to previous results with [^11^C]PBR28,^27,28^ also [^11^C]PK11195 DVR is affected by BMI and even more strongly by HOMA-IR. These similar results with two different TSPO tracers are interesting since it known that [^11^C]PBR28 and [^11^C]PK11195 have different binding properties: for instance, [^11^C]PBR28 is affected by polymorphism of the *TSPO* gene,^42^ whereas this does not seem to apply for [^11^C]PK11195. In addition, the validated analyses methods for these two tracers also differ: for [^11^C]PK11195 a supervised clustering method is used to obtain a pseudo-reference tissue for the analyses,^32^ whereas for [^11^C]PBR28 the cerebellar cortex is commonly used as a pseudo-reference region on studies focusing on AD.^43^ In contrast to our results on an association between higher BMI and higher TSPO availability, a study evaluating the role of inflammation in depression reported no association between BMI or hs-CRP and [^11^C]PK11195 binding potential (BP_ND_) in the anterior cingulate cortex, insula or the prefrontal cortex in altogether 76 individuals aged under 40 years, of whom 51 were diagnosed with depression and 25 were healthy controls.^44^ Similarly, a study with 14 depressive patients with a mean age of 30 years found no association between BMI, hs-CRP or [^11^C]PK11195 BP_ND_ in the aforementioned regions interest.^45^ These contrasting results could be explained by the differences in the age of the study populations and the studied brain regions. A previous study with [^11^C]PBR28 combining data on altogether 140 healthy control subjects from our center and two other centers indicated that age is associated with TSPO availability in the frontal and temporal cortices, but not in the total gray matter or the parietal or occipital cortices, hippocampus or thalamus.^28^ The participants of the present study were notably older than the participants of the studies focusing on depression. In addition, we evaluated associations between BMI and [^11^C]PK11195 in a cortical composite ROI consisting of wider cortical regions than the two previous studies. The previous study on [^11^C]PBR28 showed that the association between BMI and TSPO availability was stronger in men than in women.^28^ In accordance, we also detected a sex interaction for the association between HOMA-IR and TSPO availability. Here, HOMA-IR was associated with TSPO availability in men, but not in women. However, the mechanisms for these sex interactions would require further investigation.

Previous literature on the possible mechanisms on the associations between insulin resistance and TSPO availability or microglial activation is scarce. It has been suggested that neuroinflammation (referred here as the activation of microglia and the secretion of cytokines such as tumor necrosis factor-α) would be an upstream mediator of neuronal insulin resistance in the AD brain.^46^ It could be speculated that the associations we detected would be attributed to Aβ-related microglial reactivity, since we demonstrated associations in regions typical for Aβ accumulation, and the majority of our study participants were already Aβ positive. Our results indicate that there was an association between metabolic risk factors only in *APOE3/3* and *APOE4/4*, but not *APOE3/4* carriers. These findings might reflect the previously demonstrated early and late peak in neuroinflammation associated with AD pathology.^24^ However, we also found an association between HOMA-IR and TSPO binding in the occipital cortex which usually remains devoid of Aβ accumulation in AD. A previous autopsy [^3^H]PK11195 study found differences between the brains of pathologically confirmed AD cases and non-AD controls in *in vitro* [^3^H]PK11195 binding in only the parietal cortex and the hippocampus.^47^ In that study all the control cases were *APOE4* non-carriers and they did not have AD-pathology in the brain. However, it is unclear why the parietal cortex would have more TSPO availability than other regions typical for Aβ accumulation in AD. One possible explanation could be that since the parietal cortex/precuneus is one of the first regions where Aβ accumulation is seen in preclinical AD,^36^ TSPO binging in this region could reflect an inflammatory response to accumulating Aβ. It is also possible, however, that the TSPO binding that we detected could reflect non-specific binding of [^11^C]PK11195. Also the accumulation of tau pathology has been associated with TSPO PET.^48^ Tau pathology was not evaluated in the present study, and we did not find associations between metabolic risk factors and TSPO availability in regions of early tau accumulation. Considering that all the participants in our study were cognitively unimpaired we would not expect these individuals to have wide-spread tau pathology.

There are several possible ways to explain our results on an association between insulin resistance and Aβ accumulation in *APOE4/4* homozygotes. Insulin resistance, which is closely associated with obesity, has been suggested to play an important role in the AD brain.^49,50^ An attenuated response to insulin incubation by intracellular cascades triggered by the insulin receptor has been demonstrated in post mortem AD brain slices, when compared to brains of patients with MCI and cognitively normal elderly.^49^ *In vitro* and animal studies indicate that central insulin resistance/defective insulin activity could directly contribute to the accumulation of Aβ, since insulin has been shown to increase the transcription of antiamyloidogenic proteins, such as insulin degrading enzyme (IDE) which also degrades Aβ in the brain.^51^ Also, neuronal cell exposure to insulin contributes to amyloid precursor protein (APP) being processed through the nonamyloidogenic pathway, which in turn reduces Aβ40 and Aβ42 accumulation.^51^ In line with these findings, diet-induced insulin resistance in a transgenic mouse model of AD enhanced Aβ40 and Aβ42 generation and resulted in decreased IDE action in the brain.^52^ A study on ApoE targeted replacement mice indicated that ApoE4 seems to downregulate insulin receptors at the cell surface of neurons, and can therefore also contribute to impaired insulin action in the brain.^53^ Therefore, based the previous findings and ours, we propose that peripheral insulin resistance could be a risk factor for Aβ accumulation, but apparently only in *APOE4* carriers.

Our study has limitations. Imaging neuroinflammation and modelling PET data on TSPO radiotracers in living humans is challenging, and to date, no optimal tracers for imaging microglial activation exist. Moreover, a recent preprint suggested that - in humans - TSPO-targeted radiotracers reflect microglial density rather than activated microglia.^54^ Also, it is well-acknowledged that TSPO is expressed in other cell types than microglia.^23^ These methodological issues may limit the conclusions that can be drawn from our results. In addition, studies that would include also MCI and AD patients and not only cognitively unimpaired individuals would be useful to evaluate if the associations we found would be present also in regions typical for tau accumulation across the AD continuum. The strengths of this study include the well-characterized study population including equal numbers of *APOE4* non-carriers, hetero- and homozygotes which enabled the comparison between *APOE* gene doses; the multimodal imaging protocol; and correcting for partial volume effect in the [^11^C]PK11195 analyses.

In conclusion, we demonstrate that higher levels of insulin resistance and BMI are associated with higher TSPO binding in the cerebral cortex in cognitively unimpaired elderly with varying *APOE4* gene dose risk for sporadic AD. Our results suggest the risk between metabolic risk factors and an increased risk for dementia might be partly modulated through inflammatory processes in the brain, and that studies assessing TSPO availability should consider taking metabolic risk factors into account.
